# Tendon-Reflex

**Published:** 1881-02

**Authors:** 


### TENDON-REFLEX.

An important paper is contributed to *Brain* (Part X) by Dr. Augustus Waller, "On Muscular Spasms known as Tendon-Reflex." His suggestion is that the now well-known phenomenon, "patellar tendon-reflex," is not a spinal reflex, but merely a peripheral reaction of muscle, and that it is only a test of spinal conditions in the sense that other peripheral reactions of muscles (electrical or mechanical) are tests of spinal conditions. The experimental data of the paper are furnished by measurements of the interval between percussion of the tendon and contraction of the muscle. These are to the effect that on the normal human subject the above interval does not sensibly differ from the latency of the muscle to electrical excitation of its substance, or of its motor nerve; and that all these intervals, likewise the interval between percussion of the substance of a muscle and its contraction, and that also between percussion of a motor nerve and contraction of a supplied muscle, do not differ more than can be accounted for by differences in the intensity of stimulation, the normal interval or latency of all these phenomena being from 2-100 to 3-100 of a second. The paper is concerned only with the physiological interpretation of tendon-reflex as a direct "neuro-muscular" effect of the mechanical stimulus; it does not attack the clinical significance of the test.

